# Ethyl 3,4-bis­(acet­yloxy)-2-(4-meth­oxy­phen­yl)pyrrol­idine-1-carboxyl­ate

**DOI:** 10.1107/S2414314620012286

**Published:** 2020-10-30

**Authors:** Sofia Dallasta Pedroso, Ignez Caracelli, Julio Zukerman-Schpector, Monica Soto-Monsalve, Regina H. De Almeida Santos, Carlos Roque D. Correia, Ariel L. Llanes Garcia, Edward R. T. Tiekink

**Affiliations:** a Laboratório de Cristalografia, Esterodinâmica e Modelagem Molecular, Departamento de Química, Universidade Federal de São Carlos, 13565-905 São Carlos, SP, Brazil; b Departmento de Física, Universidade Federal de São Carlos, 13565-905 São Carlos, SP, Brazil; c Instituto de Química de São Carlos, Universidade de São Paulo, São Carlos, SP, Brazil; d Instituto de Química, Universidade Estadual de Campinas, UNICAMP, CP 6154, CEP 13084-917 Campinas, Brazil; e Research Centre for Crystalline Materials, School of Science and Technology, Sunway University, 47500 Bandar Sunway, Selangor Darul Ehsan, Malaysia; University of Aberdeen, Scotland

**Keywords:** crystal structure, pyrrolidine

## Abstract

The title pyrrolidine compound, C_18_H_23_NO_7_, is a tetra-substituted species in which the five-membered ring has a twisted conformation with the twist occurring in the C—C bond bearing the adjacent acet­yloxy substituents; the C_m_—C_a_—C_a_—C_p_ torsion angle is −40.76 (18)° [m = methyl­ene, a = acet­yloxy and p = phen­yl]. The N atom, which is *sp* ^2^-hybridized [sum of bond angles = 359.4°], bears an ethyl­carboxyl­ate substitutent and is connected to a methyl­ene-C atom on one side and a carbon atom bearing a 4-meth­oxy­phenyl group on the other side. Minor disorder is noted in the ethyl­carboxyl­ate substituent as well as in one of the acet­yloxy groups; the major components of the disorder have site occupancies of 0.729 (9) and 0.62 (3), respectively. The most notable feature of the mol­ecular packing is the formation of helical, supra­molecular chains aligned along the *b*-axis direction whereby the carbonyl-O atom not involved in a disordered residue accepts C—H⋯O inter­actions from methyl­ene-H and two-C atom separated methine-H atoms to form a six-membered {⋯HCCCH⋯O} synthon.

## Abstract 2

The title compound features a twisted, tetra-substituted pyrrolidine ring, and has an N-bound ethyl­carboxyl­ate substituent with the N atom flanked by a methyl­ene group on one side and a C-bound 4-meth­oxy­phenyl group on the other. These carbon atoms are linked by two methine carbon atoms, each of which bears an acet­yloxy substituent.

## Graphical abstract

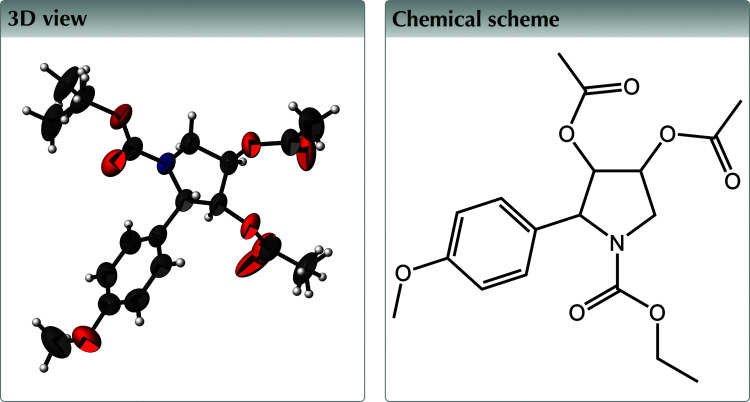

## Structure description

As reviewed recently, α-glucosidase inhibitors comprise a significant class of drugs as these are used for the treatment various disease including, among others, diabetes, cancer, cystic fibrosis and influenza (Kiappes *et al.*, 2018 ▸; Dhameja & Gupta, 2019 ▸). It was in this connection that the structure of the title tetra-substituted pyrrolidine derivative, (I), was determined in the context of supporting studies designed to provide conformational details of the mol­ecular structures of crucial synthetic inter­mediates in the generation of various α-glucosidase inhibitors (Zukerman-Schpector *et al.*, 2017 ▸; Dallasta Pedroso *et al.*, 2020*a* ▸; Dallasta Pedroso *et al.*, 2020*b* ▸).

**Figure 1:**
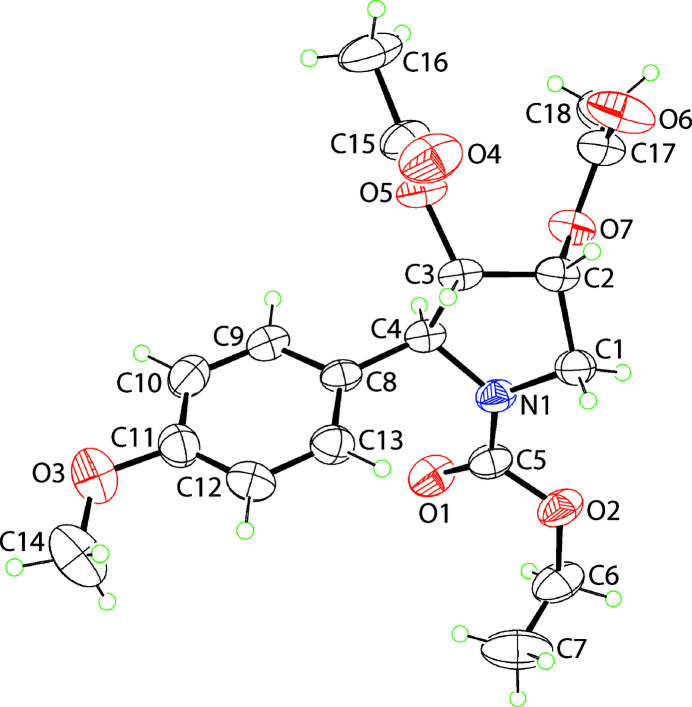
The mol­ecular structure of (I), showing the atom-labelling scheme and displacement ellipsoids at the 35% probability level. The minor components of the disordered residues are omitted.

The mol­ecular structure of (I), Fig. 1 ▸, features a five-membered pyrrolidine ring scaffold which is tetra-substituted. Thus, N1 carries a ethyl­carboxyl­ate group, each of the methine-C2 and C3 atoms carries an acet­yloxy substituent and finally, the methine-C4 atom carries a 4-meth­oxy­phenyl group. The substitution pattern indicates the presence of three chiral centres. For the illustrated mol­ecule in Fig. 1 ▸, the chirality of the C2–C4 atoms follows the sequence *R*, *S* and *S*. However, it should be noted the centrosymmetric unit cell of (I) contains equal numbers of the *S*, *R*, *R* enanti­omer. The conformation of the five-membered ring is best described as being twisted about the C2—C3 bond as seen in the value of the C1—C2—C3—C4 torsion angle of −40.76 (18)°, which is consistent with a (−)*syn*-clinal configuration. The relative orientations of the non-H substituents at the N1, C2—C4 atoms about the ring are equatorial, axial, equatorial and bis­ectional, respectively (Spek, 2020 ▸). The sum of the angles about the N1 atom comes to 359.4°, being indicative of an approximate *sp* ^2^ centre. While globally, to a first approximation, the substituents at N1 and C3 lie in the plane of the ring, the substituents at the C1 and C4 atoms lie to either side of the five-membered ring.

The substitution pattern in pyrrolidine (I) is comparatively rare with the most closely related structures being only recently reported. In one derivative, the difference arises as the N1-bound substituent is a 4-nitro­phenyl­methyl group while the other groups are the same (Dallasta Pedroso *et al.*, 2020*a* ▸) while in the other, only the substituent at the C4 differs, with the literature structure having a methyl­carboxyl­ate group (Dallasta Pedroso *et al.*, 2020*b* ▸).

**Figure 2:**
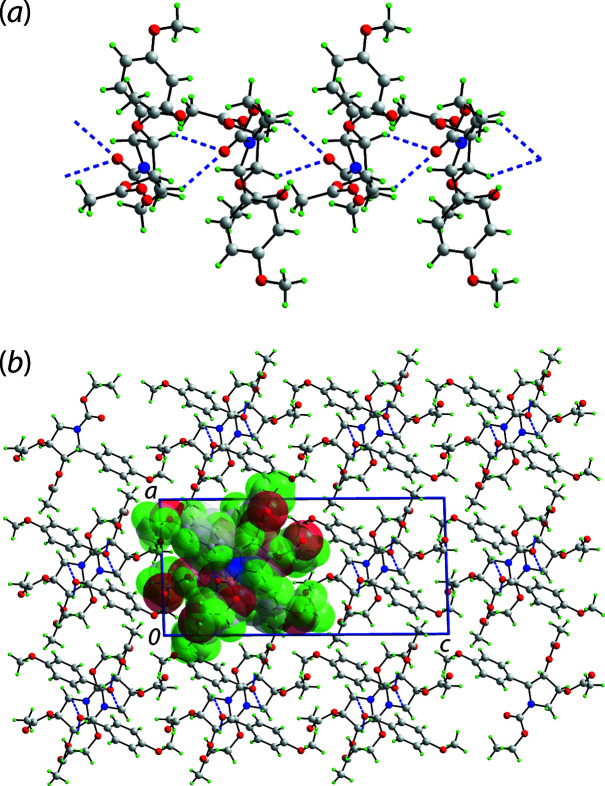
Mol­ecular packing in (I): (*a*) helical, supra­molecular chain along the *b*-axis direction sustained by C—H⋯O(carbon­yl) contacts shown as blue dashed lines and (*b*) view of the unit-cell contents shown in projection down the *b* axis, with one chain highlighted in space-filling mode.

Owing to the presence of disorder in the residues bound at the N1 and C3 atoms, a detailed analysis of the mol­ecular packing is problematic. However, supra­molecular chains propagating along the *b*-axis direction may be discerned, Fig. 2 ▸(*a*). These have a helical topology being generated by 2_1_-screw symmetry and arise as the carbonyl-O1 accepts two C—H⋯O inter­actions, Table 1 ▸, from the C1-methyl­ene and C3-methine substituents with the result that six-membered {⋯HCCCH⋯O} synthons are apparent. A view of the unit-cell contents showing the packing of chains is shown in Fig. 2 ▸(*b*).

## Synthesis and crystallization

To a solution of ethyl (2*S*,3*S*,4*R*)-3,4-dihy­droxy-2-(4-meth­oxy­phen­yl)pyrrolidine-1-carboxyl­ate (885 mg, 3.039 mmol) in CH_2_Cl_2_ (30 ml) were added pyridine (1.5 ml, 18.584 mmol), acetic anhydride (6.0 ml, 63.59 mmol) and *N*,*N*-dimethyl-4-amino­pyridine (3.7 mg, 0.030 mmol). The solution was stirred for 2 h at room temperature, concentrated in a rotary-evaporator and the residue dissolved in EtOAc (15 ml). The resulting solution was washed with a HCl 5% solution (3 × 8 ml) and with saturated solutions of NaHCO_3_ (2 × 8 ml) and of NaCl (8 ml). The phases were separated and the organic phase was dried over anhydrous Na_2_SO_4_, filtered and concentrated *in vacuo*.

The residue was purified by flash column chromatography in silica gel, using an EtOAc/*n*-hexane elution gradient (1:3 and 1:2). Yield: 1.108 g (100%). Crystals for the X-ray analysis were obtained by the slow evaporation of its *n*-hexane solution, m.p. 347–349 K.

## Refinement

Crystal data, data collection and structure refinement details are summarized in Table 2 ▸. Two residues in the mol­ecule were found to be disordered. Thus, the C7-methyl group of the N1-bound substituent was disordered over two positions, as was the carbonyl-O4 atom of the C3-acet­yloxy group. Each disorder component was refined independently and with anisotropic displacement parameters. The major components of the disorder refined to occupancies of 0.729 (9) and 0.62 (3), respectively.

## Supplementary Material

Crystal structure: contains datablock(s) I. DOI: 10.1107/S2414314620012286/hb4364sup1.cif

Structure factors: contains datablock(s) I. DOI: 10.1107/S2414314620012286/hb4364Isup2.hkl

Supporting information file. DOI: 10.1107/S2414314620012286/hb4364Isup3.cml

CCDC reference: 2027572

Additional supporting information:  crystallographic information; 3D view; checkCIF report

## Figures and Tables

**Table 1:** Hydrogen-bond geometry (Å, °)

*D*—H⋯*A*	*D*—H	H⋯*A*	*D*⋯*A*	*D*—H⋯*A*
C1—H1*B*⋯O1^i^	0.97	2.54	3.289 (2)	134
C3—H3⋯O1^i^	0.98	2.55	3.344 (2)	139

Symmetry code: (i) 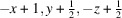 .

**Table 2:** Experimental details

Crystal data	
Chemical formula	C_18_H_23_NO_7_
*M*_r_	365.37
Crystal system, space group	Monoclinic, *P*2_1_/*c*
Temperature (K)	296
*a*, *b*, *c* (Å)	9.9429 (5), 9.3845 (5), 20.7845 (11)
β (°)	91.550 (2)
*V* (Å^3^)	1938.67 (18)
*Z*	4
Radiation type	Mo *K*α
μ (mm^−1^)	0.10
Crystal size (mm)	0.39 × 0.25 × 0.17

Data collection	
Diffractometer	Bruker APEXII CCD
Absorption correction	Multi-scan (*SADABS*; Bruker 2009 ▸)
*T*_min_, *T*_max_	0.470, 0.745
No. of measured, independent and observed [*I* > 2σ(*I*)] reflections	32581, 3970, 2612
*R*_int_	0.059
(sin θ/λ)_max_ (Å^−1^)	0.626

Refinement	
*R*[*F* ^2^ > 2σ(*F* ^2^)], *wR*(*F* ^2^), *S*	0.049, 0.140, 1.05
No. of reflections	3970
No. of parameters	261
H-atom treatment	H-atom parameters constrained
Δρ_max_, Δρ_min_ (e Å^−3^)	0.20, −0.18

Computer programs: *APEX2* and *SAINT* (Bruker, 2009 ▸), *SIR2014* (Burla *et al.*, 2015 ▸), *SHELXL2018/3* (Sheldrick, 2015 ▸), *ORTEP-3 for Windows* (Farrugia, 2012 ▸), *MarvinSketch* (ChemAxon, 2010 ▸), *DIAMOND* (Brandenburg, 2006 ▸) and *publCIF* (Westrip, 2010 ▸).

## full crystallographic data

### Crystal data

**Table:**

C_18_H_23_NO_7_	*F*(000) = 776
*M**_r_* = 365.37	*D*_x_ = 1.252 Mg m^−^^3^
Monoclinic, *P*2_1_/*c*	Mo *K*α radiation, λ = 0.71073 Å
*a* = 9.9429 (5) Å	Cell parameters from 6695 reflections
*b* = 9.3845 (5) Å	θ = 2.4–22.2°
*c* = 20.7845 (11) Å	µ = 0.10 mm^−^^1^
β = 91.550 (2)°	*T* = 296 K
*V* = 1938.67 (18) Å^3^	Slab, colourless
*Z* = 4	0.39 × 0.25 × 0.17 mm

### Data collection

**Table:**

Bruker APEXII CCD diffractometer	2612 reflections with *I* > 2σ(*I*)
φ and ω scans	*R*_int_ = 0.059
Absorption correction: multi-scan (SADABS; Bruker 2009)	θ_max_ = 26.4°, θ_min_ = 2.0°
*T*_min_ = 0.470, *T*_max_ = 0.745	*h* = −11→12
32581 measured reflections	*k* = −11→11
3970 independent reflections	*l* = −25→19

### Refinement

**Table:**

Refinement on *F*^2^	Primary atom site location: structure-invariant direct methods
Least-squares matrix: full	Secondary atom site location: difference Fourier map
*R*[*F*^2^ > 2σ(*F*^2^)] = 0.049	Hydrogen site location: inferred from neighbouring sites
*wR*(*F*^2^) = 0.140	H-atom parameters constrained
*S* = 1.05	*w* = 1/[σ^2^(*F*_o_^2^) + (0.0484*P*)^2^ + 0.6202*P*] where *P* = (*F*_o_^2^ + 2*F*_c_^2^)/3
3970 reflections	(Δ/σ)_max_ < 0.001
261 parameters	Δρ_max_ = 0.20 e Å^−^^3^
0 restraints	Δρ_min_ = −0.18 e Å^−^^3^

### Special details

**Table:**

Geometry. All esds (except the esd in the dihedral angle between two l.s. planes) are estimated using the full covariance matrix. The cell esds are taken into account individually in the estimation of esds in distances, angles and torsion angles; correlations between esds in cell parameters are only used when they are defined by crystal symmetry. An approximate (isotropic) treatment of cell esds is used for estimating esds involving l.s. planes.
Refinement. The carbon-bound H-atoms were placed in calculated positions (C—H = 0.93–0.98 Å) and were included in the refinement in the riding model approximation, with *U*_iso_(H) set to 1.2–1.5*U*_equiv_(C).

### Fractional atomic coordinates and isotropic or equivalent isotropic displacement parameters (Å^2^)

**Table:**

	*x*	*y*	*z*	*U*_iso_*/*U*_eq_	Occ. (<1)
O1	0.60299 (14)	0.86994 (16)	0.30456 (8)	0.0696 (4)	
O3	0.1472 (2)	1.0446 (2)	0.46704 (9)	0.1023 (6)	
O6	0.3095 (2)	0.9713 (3)	0.00226 (10)	0.1187 (8)	
O7	0.42775 (15)	0.89982 (16)	0.08920 (7)	0.0655 (4)	
N1	0.52014 (15)	0.99253 (17)	0.21819 (8)	0.0528 (4)	
C1	0.5423 (2)	1.0677 (2)	0.15739 (9)	0.0567 (5)	
H1A	0.620254	1.030254	0.136003	0.068*	
H1B	0.554466	1.169064	0.164517	0.068*	
C2	0.4151 (2)	1.0384 (2)	0.11882 (10)	0.0558 (5)	
H2	0.394852	1.113453	0.087240	0.067*	
C3	0.31052 (19)	1.0297 (2)	0.17042 (9)	0.0517 (5)	
H3	0.287218	1.125733	0.184930	0.062*	
C4	0.37998 (18)	0.9470 (2)	0.22556 (9)	0.0491 (5)	
H4	0.372923	0.844672	0.216805	0.059*	
C5	0.6186 (2)	0.9499 (2)	0.25960 (10)	0.0554 (5)	
O2A	0.73692 (14)	1.00916 (17)	0.24442 (8)	0.0705 (5)	0.729 (9)
C6A	0.8491 (2)	0.9829 (3)	0.28919 (14)	0.0823 (8)	0.729 (9)
H6A1	0.933380	0.995430	0.267481	0.099*	0.729 (9)
H6A2	0.845238	0.885715	0.304950	0.099*	0.729 (9)
C7A	0.8420 (6)	1.0823 (5)	0.3430 (3)	0.110 (3)	0.729 (9)
H7A1	0.842901	1.178292	0.327017	0.165*	0.729 (9)
H7A2	0.917881	1.067873	0.371778	0.165*	0.729 (9)
H7A3	0.760400	1.066240	0.365607	0.165*	0.729 (9)
O2B	0.73692 (14)	1.00916 (17)	0.24442 (8)	0.0705 (5)	0.271 (9)
C6B	0.8491 (2)	0.9829 (3)	0.28919 (14)	0.0823 (8)	0.271 (9)
H6B1	0.897975	0.898652	0.276247	0.099*	0.271 (9)
H6B2	0.816105	0.967127	0.332115	0.099*	0.271 (9)
C7B	0.9380 (13)	1.1074 (12)	0.2890 (8)	0.110 (7)	0.271 (9)
H7B1	0.953331	1.135652	0.245465	0.165*	0.271 (9)
H7B2	1.022237	1.083708	0.310020	0.165*	0.271 (9)
H7B3	0.896619	1.184482	0.311569	0.165*	0.271 (9)
C8	0.32267 (18)	0.97799 (19)	0.29014 (9)	0.0482 (5)	
C9	0.2336 (2)	0.8822 (2)	0.31699 (10)	0.0572 (5)	
H9	0.212147	0.798529	0.295049	0.069*	
C10	0.1765 (2)	0.9086 (3)	0.37533 (11)	0.0688 (6)	
H10	0.116135	0.843642	0.392146	0.083*	
C11	0.2086 (2)	1.0308 (3)	0.40881 (11)	0.0660 (6)	
C12	0.2958 (2)	1.1288 (2)	0.38331 (11)	0.0672 (6)	
H12	0.316983	1.212179	0.405547	0.081*	
C13	0.3513 (2)	1.1015 (2)	0.32416 (10)	0.0606 (5)	
H13	0.409538	1.167959	0.306848	0.073*	
C14	0.1867 (4)	1.1555 (4)	0.50790 (14)	0.1162 (11)	
H14A	0.279515	1.143361	0.520688	0.174*	
H14B	0.132295	1.154781	0.545354	0.174*	
H14C	0.175574	1.244718	0.485817	0.174*	
O4A	0.0923 (11)	1.1555 (11)	0.1225 (4)	0.094 (3)	0.62 (3)
O5A	0.19114 (13)	0.95503 (14)	0.14977 (7)	0.0627 (4)	0.62 (3)
C15A	0.0847 (2)	1.0321 (3)	0.12979 (13)	0.0698 (6)	0.62 (3)
C16A	−0.0280 (3)	0.9405 (4)	0.10542 (16)	0.1058 (10)	0.62 (3)
H16A	−0.088795	0.996594	0.079195	0.159*	0.62 (3)
H16B	−0.075114	0.901846	0.141146	0.159*	0.62 (3)
H16C	0.007324	0.864149	0.080204	0.159*	0.62 (3)
O4B	0.0711 (19)	1.1544 (18)	0.149 (2)	0.143 (8)	0.38 (3)
O5B	0.19114 (13)	0.95503 (14)	0.14977 (7)	0.0627 (4)	0.38 (3)
C15B	0.0847 (2)	1.0321 (3)	0.12979 (13)	0.0698 (6)	0.38 (3)
C16B	−0.0280 (3)	0.9405 (4)	0.10542 (16)	0.1058 (10)	0.38 (3)
H16D	−0.112233	0.984766	0.114881	0.159*	0.38 (3)
H16E	−0.022720	0.848987	0.125949	0.159*	0.38 (3)
H16F	−0.021632	0.928836	0.059714	0.159*	0.38 (3)
C17	0.3671 (3)	0.8794 (3)	0.03155 (12)	0.0788 (7)	
C18	0.3816 (3)	0.7291 (3)	0.01004 (14)	0.1128 (11)	
H18A	0.306152	0.674432	0.023937	0.169*	
H18B	0.463131	0.689770	0.028367	0.169*	
H18C	0.384959	0.726180	−0.036058	0.169*	

### Atomic displacement parameters (Å^2^)

**Table:**

	*U*^11^	*U*^22^	*U*^33^	*U*^12^	*U*^13^	*U*^23^
O1	0.0625 (9)	0.0584 (9)	0.0870 (11)	0.0014 (7)	−0.0158 (8)	0.0222 (8)
O3	0.1082 (15)	0.1215 (16)	0.0783 (12)	0.0018 (12)	0.0220 (11)	−0.0096 (12)
O6	0.143 (2)	0.1322 (19)	0.0783 (13)	0.0106 (15)	−0.0538 (13)	−0.0073 (12)
O7	0.0731 (10)	0.0659 (9)	0.0566 (8)	−0.0001 (7)	−0.0147 (7)	−0.0110 (7)
N1	0.0451 (9)	0.0578 (10)	0.0548 (10)	−0.0031 (7)	−0.0115 (7)	0.0042 (8)
C1	0.0545 (12)	0.0601 (12)	0.0549 (12)	−0.0054 (9)	−0.0077 (9)	0.0005 (10)
C2	0.0628 (12)	0.0494 (11)	0.0544 (11)	−0.0025 (9)	−0.0142 (9)	0.0007 (9)
C3	0.0489 (11)	0.0449 (10)	0.0602 (12)	−0.0030 (8)	−0.0167 (9)	−0.0027 (9)
C4	0.0484 (10)	0.0419 (10)	0.0562 (11)	−0.0016 (8)	−0.0112 (9)	0.0001 (8)
C5	0.0511 (11)	0.0462 (11)	0.0680 (13)	0.0012 (9)	−0.0132 (10)	0.0013 (10)
O2A	0.0485 (8)	0.0791 (10)	0.0827 (11)	−0.0087 (7)	−0.0197 (7)	0.0185 (8)
C6A	0.0513 (13)	0.0856 (17)	0.108 (2)	0.0014 (12)	−0.0293 (13)	0.0188 (16)
C7A	0.111 (4)	0.087 (3)	0.128 (4)	0.018 (3)	−0.070 (4)	−0.013 (3)
O2B	0.0485 (8)	0.0791 (10)	0.0827 (11)	−0.0087 (7)	−0.0197 (7)	0.0185 (8)
C6B	0.0513 (13)	0.0856 (17)	0.108 (2)	0.0014 (12)	−0.0293 (13)	0.0188 (16)
C7B	0.070 (8)	0.090 (8)	0.165 (14)	−0.003 (6)	−0.069 (9)	0.013 (8)
C8	0.0443 (10)	0.0444 (10)	0.0550 (11)	0.0012 (8)	−0.0132 (8)	0.0024 (8)
C9	0.0505 (11)	0.0522 (12)	0.0682 (13)	−0.0048 (9)	−0.0091 (10)	0.0006 (10)
C10	0.0554 (13)	0.0735 (15)	0.0774 (15)	−0.0079 (11)	0.0009 (11)	0.0085 (12)
C11	0.0610 (13)	0.0792 (16)	0.0576 (13)	0.0100 (12)	0.0009 (11)	0.0034 (12)
C12	0.0784 (15)	0.0598 (13)	0.0627 (14)	0.0030 (11)	−0.0107 (12)	−0.0080 (11)
C13	0.0685 (13)	0.0491 (11)	0.0639 (13)	−0.0068 (10)	−0.0045 (10)	−0.0005 (10)
C14	0.163 (3)	0.108 (2)	0.0780 (19)	0.025 (2)	0.014 (2)	−0.0093 (18)
O4A	0.077 (4)	0.077 (4)	0.125 (5)	0.006 (3)	−0.035 (3)	0.032 (4)
O5A	0.0529 (8)	0.0548 (8)	0.0789 (10)	−0.0057 (6)	−0.0255 (7)	−0.0020 (7)
C15A	0.0505 (13)	0.0730 (16)	0.0848 (17)	−0.0012 (11)	−0.0194 (11)	0.0147 (14)
C16A	0.0675 (16)	0.120 (2)	0.128 (2)	−0.0181 (16)	−0.0458 (17)	0.011 (2)
O4B	0.062 (5)	0.065 (6)	0.30 (2)	0.019 (4)	−0.034 (11)	−0.042 (10)
O5B	0.0529 (8)	0.0548 (8)	0.0789 (10)	−0.0057 (6)	−0.0255 (7)	−0.0020 (7)
C15B	0.0505 (13)	0.0730 (16)	0.0848 (17)	−0.0012 (11)	−0.0194 (11)	0.0147 (14)
C16B	0.0675 (16)	0.120 (2)	0.128 (2)	−0.0181 (16)	−0.0458 (17)	0.011 (2)
C17	0.0763 (16)	0.098 (2)	0.0609 (15)	−0.0115 (14)	−0.0149 (12)	−0.0149 (14)
C18	0.131 (3)	0.113 (2)	0.094 (2)	−0.020 (2)	−0.0115 (18)	−0.0440 (19)

### Geometric parameters (Å, º)

**Table:**

O1—C5	1.212 (2)	C7B—H7B1	0.9600
O3—C11	1.376 (3)	C7B—H7B2	0.9600
O3—C14	1.393 (4)	C7B—H7B3	0.9600
O6—C17	1.193 (3)	C8—C13	1.383 (3)
O7—C17	1.341 (3)	C8—C9	1.389 (3)
O7—C2	1.446 (2)	C9—C10	1.375 (3)
N1—C5	1.347 (2)	C9—H9	0.9300
N1—C1	1.469 (3)	C10—C11	1.375 (3)
N1—C4	1.469 (2)	C10—H10	0.9300
C1—C2	1.504 (3)	C11—C12	1.380 (3)
C1—H1A	0.9700	C12—C13	1.385 (3)
C1—H1B	0.9700	C12—H12	0.9300
C2—C3	1.516 (3)	C13—H13	0.9300
C2—H2	0.9800	C14—H14A	0.9600
C3—O5B	1.434 (2)	C14—H14B	0.9600
C3—O5A	1.434 (2)	C14—H14C	0.9600
C3—C4	1.533 (3)	O4A—C15A	1.171 (10)
C3—H3	0.9800	O5A—C15A	1.338 (2)
C4—C8	1.501 (3)	C15A—C16A	1.490 (3)
C4—H4	0.9800	C16A—H16A	0.9600
C5—O2B	1.346 (3)	C16A—H16B	0.9600
C5—O2A	1.346 (3)	C16A—H16C	0.9600
O2A—C6A	1.454 (2)	O4B—C15B	1.225 (19)
C6A—C7A	1.460 (5)	O5B—C15B	1.338 (2)
C6A—H6A1	0.9700	C15B—C16B	1.490 (3)
C6A—H6A2	0.9700	C16B—H16D	0.9600
C7A—H7A1	0.9600	C16B—H16E	0.9600
C7A—H7A2	0.9600	C16B—H16F	0.9600
C7A—H7A3	0.9600	C17—C18	1.488 (4)
O2B—C6B	1.454 (2)	C18—H18A	0.9600
C6B—C7B	1.466 (12)	C18—H18B	0.9600
C6B—H6B1	0.9700	C18—H18C	0.9600
C6B—H6B2	0.9700		
			
C11—O3—C14	118.8 (2)	H7B1—C7B—H7B2	109.5
C17—O7—C2	117.81 (19)	C6B—C7B—H7B3	109.5
C5—N1—C1	124.68 (17)	H7B1—C7B—H7B3	109.5
C5—N1—C4	121.50 (16)	H7B2—C7B—H7B3	109.5
C1—N1—C4	113.18 (14)	C13—C8—C9	117.48 (19)
N1—C1—C2	103.15 (16)	C13—C8—C4	122.78 (18)
N1—C1—H1A	111.1	C9—C8—C4	119.71 (17)
C2—C1—H1A	111.1	C10—C9—C8	121.4 (2)
N1—C1—H1B	111.1	C10—C9—H9	119.3
C2—C1—H1B	111.1	C8—C9—H9	119.3
H1A—C1—H1B	109.1	C11—C10—C9	120.1 (2)
O7—C2—C1	108.04 (16)	C11—C10—H10	120.0
O7—C2—C3	108.80 (16)	C9—C10—H10	120.0
C1—C2—C3	102.45 (15)	C10—C11—O3	114.9 (2)
O7—C2—H2	112.3	C10—C11—C12	120.1 (2)
C1—C2—H2	112.3	O3—C11—C12	125.0 (2)
C3—C2—H2	112.3	C11—C12—C13	119.2 (2)
O5B—C3—C2	113.11 (15)	C11—C12—H12	120.4
O5A—C3—C2	113.11 (15)	C13—C12—H12	120.4
O5B—C3—C4	109.21 (15)	C8—C13—C12	121.8 (2)
O5A—C3—C4	109.21 (15)	C8—C13—H13	119.1
C2—C3—C4	104.62 (15)	C12—C13—H13	119.1
O5A—C3—H3	109.9	O3—C14—H14A	109.5
C2—C3—H3	109.9	O3—C14—H14B	109.5
C4—C3—H3	109.9	H14A—C14—H14B	109.5
N1—C4—C8	114.91 (15)	O3—C14—H14C	109.5
N1—C4—C3	100.63 (15)	H14A—C14—H14C	109.5
C8—C4—C3	113.36 (16)	H14B—C14—H14C	109.5
N1—C4—H4	109.2	C15A—O5A—C3	118.03 (16)
C8—C4—H4	109.2	O4A—C15A—O5A	121.4 (5)
C3—C4—H4	109.2	O4A—C15A—C16A	125.3 (5)
O1—C5—O2B	124.58 (18)	O5A—C15A—C16A	112.0 (2)
O1—C5—O2A	124.58 (18)	C15A—C16A—H16A	109.5
O1—C5—N1	124.77 (19)	C15A—C16A—H16B	109.5
O2B—C5—N1	110.65 (18)	H16A—C16A—H16B	109.5
O2A—C5—N1	110.65 (18)	C15A—C16A—H16C	109.5
C5—O2A—C6A	116.09 (17)	H16A—C16A—H16C	109.5
O2A—C6A—C7A	109.3 (2)	H16B—C16A—H16C	109.5
O2A—C6A—H6A1	109.8	C15B—O5B—C3	118.03 (16)
C7A—C6A—H6A1	109.8	O4B—C15B—O5B	119.9 (10)
O2A—C6A—H6A2	109.8	O4B—C15B—C16B	124.3 (8)
C7A—C6A—H6A2	109.8	O5B—C15B—C16B	112.0 (2)
H6A1—C6A—H6A2	108.3	C15B—C16B—H16D	109.5
C6A—C7A—H7A1	109.5	C15B—C16B—H16E	109.5
C6A—C7A—H7A2	109.5	H16D—C16B—H16E	109.5
H7A1—C7A—H7A2	109.5	C15B—C16B—H16F	109.5
C6A—C7A—H7A3	109.5	H16D—C16B—H16F	109.5
H7A1—C7A—H7A3	109.5	H16E—C16B—H16F	109.5
H7A2—C7A—H7A3	109.5	O6—C17—O7	123.4 (2)
C5—O2B—C6B	116.09 (17)	O6—C17—C18	125.5 (2)
O2B—C6B—C7B	108.4 (4)	O7—C17—C18	111.0 (3)
O2B—C6B—H6B1	110.0	C17—C18—H18A	109.5
C7B—C6B—H6B1	110.0	C17—C18—H18B	109.5
O2B—C6B—H6B2	110.0	H18A—C18—H18B	109.5
C7B—C6B—H6B2	110.0	C17—C18—H18C	109.5
H6B1—C6B—H6B2	108.4	H18A—C18—H18C	109.5
C6B—C7B—H7B1	109.5	H18B—C18—H18C	109.5
C6B—C7B—H7B2	109.5		
			
C5—N1—C1—C2	158.14 (18)	C5—O2A—C6A—C7A	−81.8 (4)
C4—N1—C1—C2	−12.8 (2)	O1—C5—O2B—C6B	−5.5 (3)
C17—O7—C2—C1	−147.52 (19)	N1—C5—O2B—C6B	174.16 (18)
C17—O7—C2—C3	102.0 (2)	C5—O2B—C6B—C7B	−147.9 (8)
N1—C1—C2—O7	−82.60 (19)	N1—C4—C8—C13	38.0 (2)
N1—C1—C2—C3	32.16 (19)	C3—C4—C8—C13	−77.0 (2)
O7—C2—C3—O5B	−45.3 (2)	N1—C4—C8—C9	−144.04 (17)
C1—C2—C3—O5B	−159.51 (15)	C3—C4—C8—C9	101.0 (2)
O7—C2—C3—O5A	−45.3 (2)	C13—C8—C9—C10	−0.3 (3)
C1—C2—C3—O5A	−159.51 (15)	C4—C8—C9—C10	−178.40 (18)
O7—C2—C3—C4	73.45 (17)	C8—C9—C10—C11	−0.9 (3)
C1—C2—C3—C4	−40.76 (18)	C9—C10—C11—O3	−178.9 (2)
C5—N1—C4—C8	54.7 (2)	C9—C10—C11—C12	1.4 (3)
C1—N1—C4—C8	−134.09 (17)	C14—O3—C11—C10	171.9 (2)
C5—N1—C4—C3	176.83 (17)	C14—O3—C11—C12	−8.5 (4)
C1—N1—C4—C3	−11.9 (2)	C10—C11—C12—C13	−0.7 (3)
O5B—C3—C4—N1	153.37 (15)	O3—C11—C12—C13	179.6 (2)
O5A—C3—C4—N1	153.37 (15)	C9—C8—C13—C12	1.0 (3)
C2—C3—C4—N1	32.01 (18)	C4—C8—C13—C12	179.05 (18)
O5B—C3—C4—C8	−83.41 (19)	C11—C12—C13—C8	−0.5 (3)
O5A—C3—C4—C8	−83.41 (19)	C2—C3—O5A—C15A	−98.4 (2)
C2—C3—C4—C8	155.23 (15)	C4—C3—O5A—C15A	145.5 (2)
C1—N1—C5—O1	−168.3 (2)	C3—O5A—C15A—O4A	8.2 (7)
C4—N1—C5—O1	1.8 (3)	C3—O5A—C15A—C16A	175.8 (2)
C1—N1—C5—O2B	12.0 (3)	C2—C3—O5B—C15B	−98.4 (2)
C4—N1—C5—O2B	−177.78 (16)	C4—C3—O5B—C15B	145.5 (2)
C1—N1—C5—O2A	12.0 (3)	C3—O5B—C15B—O4B	−25 (2)
C4—N1—C5—O2A	−177.78 (16)	C3—O5B—C15B—C16B	175.8 (2)
O1—C5—O2A—C6A	−5.5 (3)	C2—O7—C17—O6	3.9 (4)
N1—C5—O2A—C6A	174.16 (18)	C2—O7—C17—C18	−176.0 (2)

### Hydrogen-bond geometry (Å, º)

**Table:**

*D*—H···*A*	*D*—H	H···*A*	*D*···*A*	*D*—H···*A*
C1—H1*B*···O1^i^	0.97	2.54	3.289 (2)	134
C3—H3···O1^i^	0.98	2.55	3.344 (2)	139

Symmetry code: (i) −*x*+1, *y*+1/2, −*z*+1/2.
